# Normal and shear strains of the left ventricle in healthy human subjects measured by two-dimensional speckle tracking echocardiography

**DOI:** 10.1186/1476-7120-12-7

**Published:** 2014-02-11

**Authors:** Li-Jun Yuan, Katsu Takenaka, Kansei Uno, Aya Ebihara, Kazuno Sasaki, Takako Komuro, Makoto Sonoda, Ryozo Nagai

**Affiliations:** 1 Department of Ultrasound Diagnostics, Tangdu Hospital, Fourth Military Medical University, Xi’an 710038, China; 2 Department of Laboratory Medicine, University of Tokyo Hospital, Tokyo 1138655, Japan; 3 Department of Cardiovascular Medicine, University of Tokyo, 7-3-1 Hongo, Bunkyo, Tokyo 113-8655, Japan

**Keywords:** 2-D speckle tracking, Strain, Left ventricle, Echocardiography

## Abstract

**Background:**

Animal studies have shown that shear deformation of myocardial sheets in transmural planes of left ventricular (LV) wall is an important mechanism for systolic wall thickening, and normal and shear strains of the LV free wall differ from those of the interventricular septum (IVS). We sought to test whether these also hold for human hearts.

**Methods:**

Thirty healthy volunteers (male 23 and female 7, aged 34 ± 6 years) from Outpatient Department of the University of Tokyo Hospital were included. Echocardiographic images were obtained in the left decubitus position using a commercially available system (Aloka SSD-6500, Japan) equipped with a 3.5-MHz transducer. The ECG was recorded simultaneously. The peak systolic radial normal strain (length change), shear strain (angle change) and time to peak systolic radial normal strain were obtained non-invasively by two-dimensional speckle tracking echocardiography.

**Results:**

The peak systolic radial normal strain in both IVS and LV posterior wall (LVPW) showed a trend to increase progressively from the apical level to the basal level, especially at short axis views, and the peak systolic radial normal strain of LVPW was significantly greater than that of IVS at all three levels. The time to peak systolic radial normal strain was the shortest at the basal IVS, and increased progressively from the base to the apical IVS. It gradually increased from the apical to the basal LVPW in sequence, especially at short axis views. The peak of radial normal strain of LVPW occurred much later than the peak of IVS at all three levels. For IVS, the shear deformation was clockwise at basal level, and counterclockwise at mid and apical levels in LV long-axis view. For LVPW, the shear deformations were all counterclockwise in LV long-axis view and increased slightly from base to the apex. LVPW showed larger shear strains than IVS at all three levels. Bland-Altman analysis shows very good agreement between measurements taken by the same observer and by two independent observers.

**Conclusion:**

“Myocardial sheets” theory also holds true for intact human LV. Moreover, dyssynchrony exists even in healthy human subjects, which should be considered when evaluating the diseased hearts.

## Introduction

Assessment of regional left ventricular (LV) function is essential for the evaluation and management of patients with heart disease, and it is of great importance to know how the normal LV behaves before we make a diagnosis of cardiac dysfunction. Newly developed two-dimensional (2D) speckle tracking echocardiography based on tracking of the speckles produced by the interaction of ultrasound with the ventricular structures has made the quantification of LV regional function more accurate and simpler compared to the other techniques [1-4].

**Figure 1:**
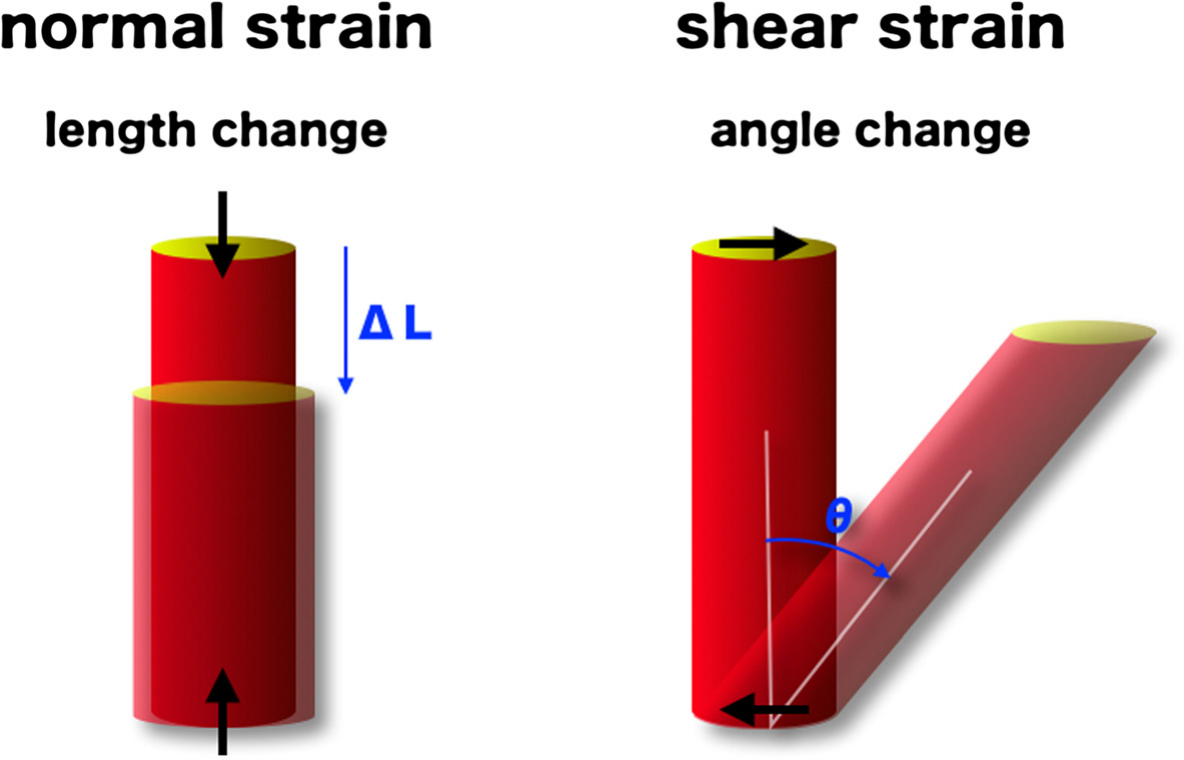
Scheme indicating the normal and shear strain.

Using sonomicrometry and magnetic resonance imaging tagging as reference methods, a number of researchers verified that speckle tracking echocardiography could provide accurate measurement of LV deformation [5-9]. Relative amount of deformation is defined as strain. In a one-dimensional object, the only possible deformation of the object is lengthening or shortening, which is referred to as normal strain (Figure 1); for a two- or three-dimensional object, it is not just limited to the lengthening and shortening that is normal (perpendicular) to the border of the object, but also involved the motion that is parallel to the border of the object, which is called the shear strain (Figure 1). Myocardium, as a three dimensional object, has three normal strains along three axes (x, y and z; i.e., radial, longitudinal and circumferential, respectively) and six (y-x, z-y and z-x; i.e., longitudinal-radial, circumferential-longitudinal and circumferential-radial, respectively) shear strains along six planes [10]. Note there are some other ways to describe strains. When the length of the object is not only known before and after deformation but also during the deformation process, it is called instantaneous strain. When the instantaneous deformation is expressed relative to the initial length, it is called Lagrangian strain; if the reference value is not constant over time but changes during the deformation process, it is called natural strain [11].

Recent descriptions of the laminar organization of myocardium have provided a possible structural link between transmural shear and wall thickening [10,12]. It has been shown that myocardium to be laminar in nature, with sheets of myocytes (on average, four cells thick) connected by a loose collagen network that spans the cleavage planes between the sheets [10]. Animal studies by cineangiographic evaluation of metalic beads implanted directly into the ventricular wall have shown that shear deformation (shear strain) of myocardial sheets in transmural planes of LV wall is an important mechanism for systolic wall thickening [10,12]. There are significant regional variations in the organization of the myocardial sheets, the differences being particularly marked between the subendocardial regions of the LV free wall and the interventricular septum of the canine heart. In the LV free wall of the canine heart, the myocardial sheets curve steeply toward the basal direction as they approach the endocardium and become nearly parallel to the endocardial surface, whereas in the septum, the myocardial sheets curve less steeply toward the apical direction as they approach the endocardium and do not become nearly parallel to the endocardial surface, resulting in the difference of shear strain and radial strain between the LV free walls (LVFW) and the interventricular septum (IVS) (Additional file 1) [10,12]. We hypothesize this is also true in healthy human subjects.

To test the hypothesis, we evaluated the radial normal strain, which refers to the wall thickness change in the radial direction perpendicular to the endocardium of a given segment, and shear strain, which refers to the angle change of the line between the endocardium and epicardium of a given segment, in the LV free wall (LVPW) and IVS of healthy subjects, with 2D speckle tracking echocardiography.

## Methods

### Study population

Thirty healthy volunteers (male 23 and female 7, aged 34 ± 6 years) from Outpatient Department of the University of Tokyo Hospital were included and proved to have no cardiopulmonary diseases by inquiry, physical examination, blood pressure measurement, electrocardiogram, echocardiogram, and lung function examination. The study protocol was approved by the Committee of University of Tokyo Hospital. All participants gave informed consent to attend this study.

### Echocardiography

#### Equipment

Echocardiographic images were obtained in the left decubitus position using a commercially available system (Aloka SSD-6500, Japan) equipped with a 3.5-MHz transducer. The ECG was recorded simultaneously. Images were acquired in cine loops triggered to the QRS complex and saved in digital format to a magneto-optical disk for off-line analysis.

#### Echocardiographic recordings and analyses

Two-dimensional echocardiographic parasternal long-axis view recorded at a frame rate of 60–70 frames/s were obtained using standard parasternal and modified apical LV long-axis views at basal, mid and apical levels. The modified apical LV long-axis view was acquired by slightly tilting towards the apex from the standard parasternal LV long-axis view. The spatial resolution is less than 3 mm. The basal level was defined as the position just below the mitral leaflet, the mid level was defined as the place of the papillary muscle, and the apical level was defined as the position below the papillary muscle.

**Figure 2:**
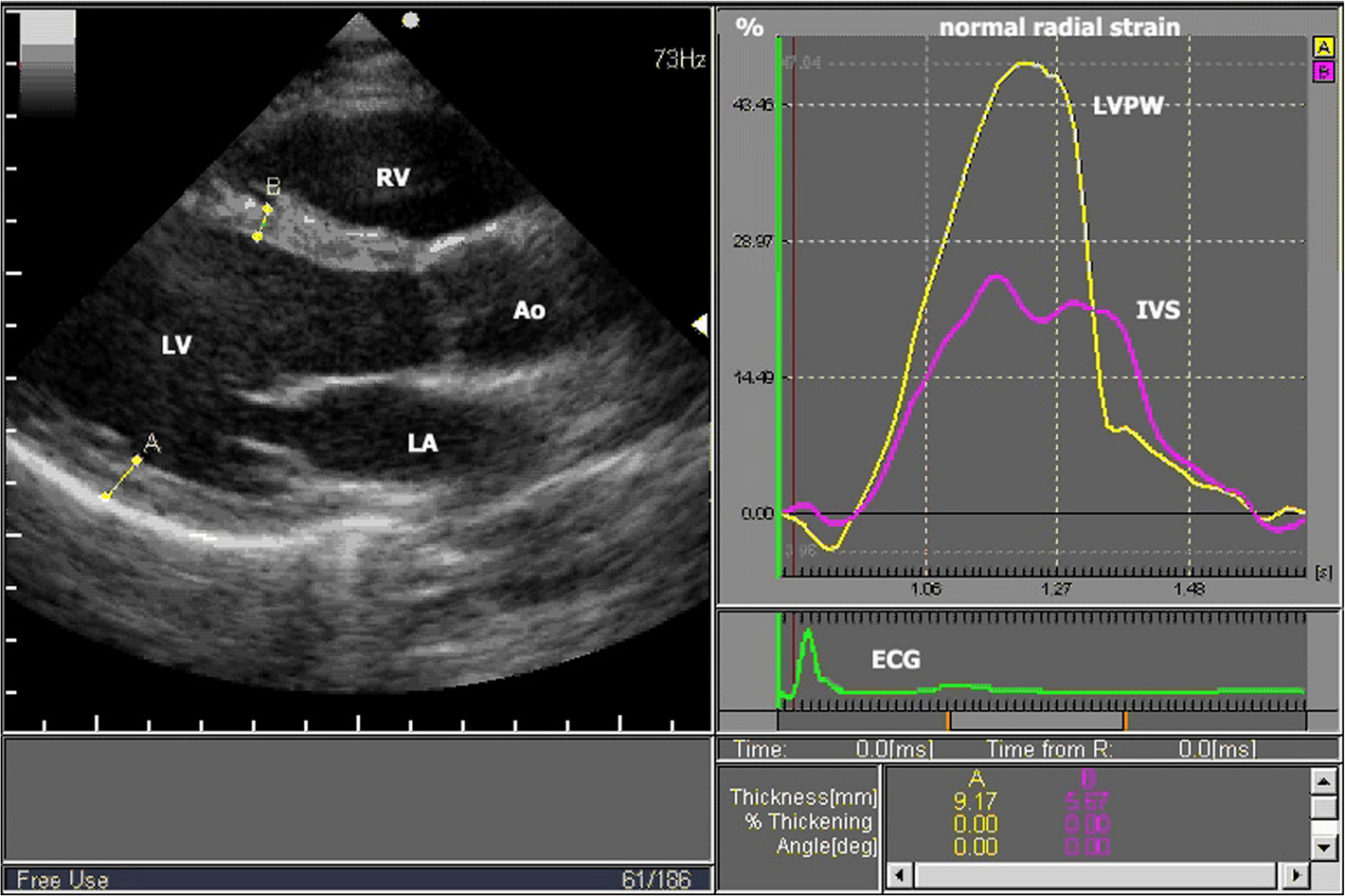
**Measurement of radial normal strain of IVS and LVPW at mid level in parasternal LV long axis view (taken from Additional file****2****).** Right panel shows radial normal strain curves for left ventricular posterior wall (LVPW) and interventricular septum (IVS). IVS strain peaks earlier than LVPW strain, and peak strain value is about 2 times larger in LVPW than in IVS. Ao = aorta, ECG = electrocardiogram, LA = left atrium, LV = left ventricle, and RV = right ventricle.

From an end-systolic single frame of the long-axis view, 2 points were designated at the basal , mid, and apical levels in the IVS (the LV-side endocardium and the RV-side endocardium) and corresponding 2 points was designated at the same levels in the LVPW (the endocardium and the epicardium) by a point-and-draw approach (Figure 2 and Additional file 2). The basal level was defined as the position just below the mitral leaflet, the mid level was defined as the level of the papillary muscle, and the apical level was defined as the position below the papillary muscle. Acoustic markers, the so-called speckles, equally distributed in the region of interest, could be followed throughout the entire cardiac cycle and parameters of myocardial deformation could be calculated by clicking processing button. In order to corresponding with the laminar structure orientation of the heart displayed in the study by LeGrice IJ and to verify the “myocardial sheets” theory also holds for intact human LV, endocardial to epicardial boarders were traced.

In this study, the peak systolic radial normal strain (the maximal wall thickening during systole), shear strain (the angle between the sheared line and its original line) and time to peak systolic radial normal strain (time from the onset of QRS to the time peak systolic radial strain occurs, ms) were obtained (Figure 2). Each value represented the average of three repeated measurements. The myocardial thickening deformation and counter-clockwise shear angle were defined as positive strain.

Routine two-dimensional and Doppler echocardiography were performed and the ejection fraction (EF), fractional shortening (FS) and E/A ratio were obtained in accordance with the recommendations of the American Society of Echocardiography Committee [13].

### Inter- and intra-observer variability

To assess inter- and intra-observer variability, ten echocardiographic recordings were randomly selected and then the measurements difference in peak radial normal strain and time to peak systolic normal radial strain were analyzed.

### Statistical analysis

Data were expressed as mean ± SD and are analyzed with paired t-test between LVPW and IVS. Statistical differences are considered significant at P < 0.05.

## Results

**Figure 3:**
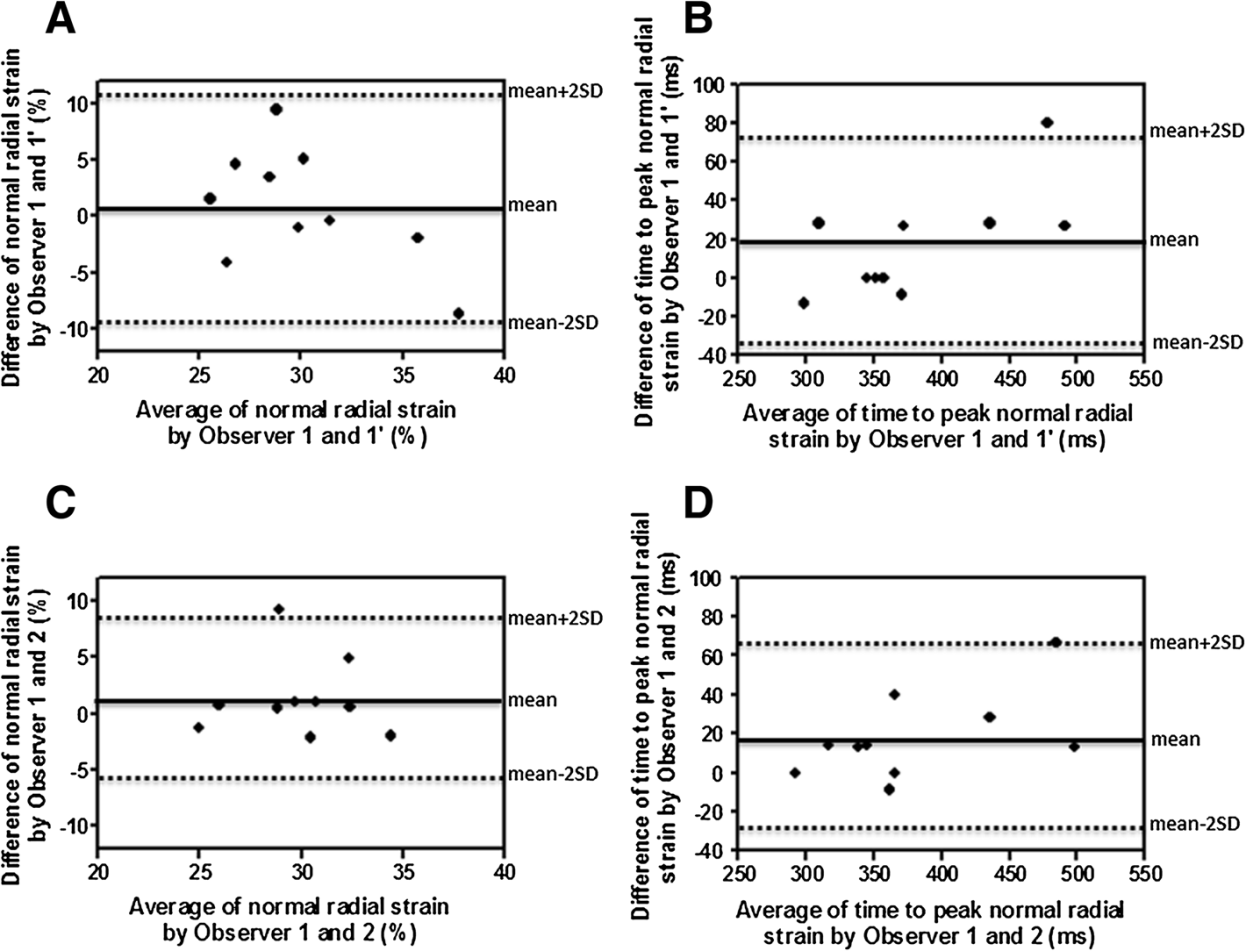
**Intra- and inter-observer variability for normal radial strain and time to peak systolic normal radial strain.** Bland-Altman analysis shows very good agreement between measurements taken by the same observer **(A, B)** and by two independent observers **(C, D)**.

1. **LV dimensions and systolic function:** LV end-diastolic and end-systolic dimensions, EF, FS and E/A ratio of 30 study subjects were shown in Table 1.

2. **Peak systolic radial normal strain:** Peak systolic radial normal strain in both IVS and LVPW showed a trend to increase progressively from the apical level to the basal level, especially at short axis views, and the peak systolic radial normal strain of LVPW was significantly greater than that of IVS at all three levels (Tables 2, 3; Additional file 2).

3. **Time to peak systolic radial normal strain:** Time to peak systolic radial normal strain was the shortest at the basal IVS, and increased progressively from the base to the apical IVS. It gradually increased from the apical to the basal LVPW in sequence, especially at short axis views. The peak of radial normal strain of LVPW occurred much later than the peak of IVS at all three levels (Tables 2, 3; Additional file 2).

4. **Shear strain:** For IVS, the shear deformation was clockwise at basal level, and counterclockwise at mid and apical levels in LV long-axis view. For LVPW, the shear deformations were all counterclockwise in LV long-axis view and increased slightly from base to the apex. LVPW showed larger shear strains than IVS at all three levels (Table 2, Additional files 1 and 2). No regular changes were found in shear strain at LV short-axis views. These results correspond to the fact that myocardial sheets are most clearly recognized on the LV long-axis section. There was no significant difference at the 3 levels within the same wall. However, significant difference was found in time-to-peak strain at the 3 levels within the same wall (p < 0.05) (Table 4).

5. **Inter- and intra-observer variability:** Bland-Altman analysis shows very good agreement between measurements taken by the same observer (0.80 ± 5.17% for peak normal radial strain and 16.7 ± 27.5 ms for time to peak systolic radial normal strain) (Figure 3A and B) and by two independent observers (1.25 ± 3.43% for peak normal radial strain and 18.0 ± 22.0 ms for time to peak systolic radial normal strain) (Figure 3C and D).

**Table 1:** Traditional echocardiographic parameters of the study population (mean ± SD)

**IVS (cm)**	**LVPW (cm)**	**LVEDd (cm)**	**LVEDs (cm)**	**SV (ml)**	**EF (%)**	**FS (%)**	**E/A ratio**
0.73 ± 0.10	0.66 ± 0.11	4.97 ± 0.31	3.05 ± 0.26	81.0 ± 16.70	68.2 ± 7.1	38.6 ± 5.3	1.55 ± 0.11

Notes: IVS, Interventricular septum; LVPW, Left ventricular posterior wall; LVEDd, Left ventricular end-diastolic dimension; LVEDs, Left ventricular end-systolic dimension; SV, stroke volume; EF, Ejection fraction; FS, Fractional shortening.

**Table 2:** Peak systolic radial normal strain, time to peak systolic radial normal strain and shear strain of LVPW, IVS in parasternal long axis views (mean ± SD)

**Variance**	**Wall**	**Base**	**Mid**	**Apex**
**Normal peak systolic radial strain**	**IVS**	0.33 ± 0.13	0.35 ± 0.15	0.31 ± 0.16
	**LVPW**	0.49 ± 0.16	0.50 ± 0.16	0.43 ± 0.16
	**P**	<0.00001	<0.001	<0.001
**Time to peak systolic radial strain (ms)**	**IVS**	342 ± 34	333 ± 25	342 ± 24
	**LVPW**	413 ± 36^***^^&&^	384 ± 38	384 ± 45
	**P**	<0.00001	<0.00001	<0.00001
**Shear strain (degree)**	**IVS**	−6.8 ± 7.9	2.0 ± 6.7	6.0 ± 7.6
	**LVPW**	7.5 ± 11.9	8.6 ± 10.6	11.6 ± 9.6
	**P**	<0.00001	<0.05	<0.05

^***^Compared to the apex of the same wall, P < 0.001; ^&&^Compared to the mid of the same wall, P < 0.01. LVPW, Left ventricular posterior wall; IVS, Interventricular septum; LV, Left ventricle.

**Table 3:** Peak systolic radial normal strain, time to peak systolic radial normal strain and shear strain of LVPW, IVS in parasternal short axis views (mean ± SD)

**Variance**	**Wall**	**Base**	**Mid**	**Apex**
**Normal peak systolic radial strain**	**IVS**	0.37 ± 0.11^*^	0.32 ± 0.15^**^	0.25 ± 0.11
	**LVPW**	0.59 ± 0.18	0.51 ± 0.16	0.35 ± 0.14^***^
	**P**	<0.00001	<0.00001	<0.01
**Time to peak systolic radial strain (ms)**	**IVS**	341 ± 35^*^	343 ± 23^*^	361 ± 35
	**LVPW**	413 ± 43^*^^&^	384 ± 37	385 ± 47
	**P**	<0.00001	<0.00001	<0.01
**Shear strain (degree)**	**IVS**	−2.1 ± 9.1	0.0 ± 6.2	2.5 ± 8.6
	**LVPW**	−1.0 ± 9.4	−0.1 ± 9.0	1.3 ± 8.8
	**P**	ns	ns	ns

Notes: ^*^Compared to the apex of the same wall, P < 0.05; ^**^Compared to the apex of the same wall, P < 0.01; ^***^Compared to the apex of the same wall, P < 0.0001; ^&^Compared to the mid of the same wall, P < 0.01. LVPW, Left ventricular posterior wall; IVS, Interventricular septum; LV, Left ventricle.

**Table 4:** **Comparison of peak systolic radial normal strain and time to peak systolic radial normal strain at 3 levels within the same wall (*p *** **values)**

	**LAX**					**SAX**		
	**Radial normal strain**		**Time to peak normal radial strain**		**Radial normal strain**		**Time to peak normal radial strain**	
	**IVS**	**LVPW**	**IVS**	**LVPW**	**IVS**	**LVPW**	**IVS**	**LVPW**
**Base-Mid**	0.44	0.95	0.23	0.0027	0.10	0.05	0.62	0.005
**Base-Apex**	0.96	0.10	0.98	0.0003	0.0004	<0.0001	0.014	0.013
**Mid-Apex**	0.45	0.07	0.10	0.95	0.02	0.0006	0.002	0.901

Notes: LAX, long-axis view of LV; SAX, short-axis view; LVPW, Left ventricular posterior wall; IVS, Interventricular septum; LV, Left ventricle.

## Discussion

This study showed that the “myocardial sheets” theory was also applicable in healthy human subjects, and significant differences of peak systolic radial normal strain, time to peak systolic radial normal strain as well as shear strain existed between LVPW and IVS even in healthy human subjects.

### Mechanism of LV wall thickening

According to law of conservation of mass, shortening in longitudinal and circumferential direction should result in thickening in the radial direction. In this study, we analyzed the radial normal strain (segmental length change in radial direction), which are actually the systolic wall thickenings. As reported by LeGrice et al. and illustrated in Additional file 1, when myocardial cells contract, the myocardial sheets slide on each other causing shear strain, which is thought to play an important role in the mechanical function of the LV [12]. Since each myocyte shortens only 10% in its longitudinal direction and thickens only 10% in its transverse direction, it is clear that the complexity of fiber direction is a prerequisite to radial wall thickening of 50% to 70% of LVPW [8,14]. In other words, the systolic wall thickening is not caused by simple thickening of individual myocytes in concert but also by the effects of shearing motion of groups of myocytes (myocardial sheets) on each other.

Despite the evident importance of shear deformation in normal LV function, little direct information is available about the shear properties of human LV wall. In animal studies, it showed that the myocardial sheets or cleavage planes approached the LV endocardium obliquely from the apical direction, becoming nearly parallel to the endocardial surface in LVPW while, in the IVS, the sheets approached the LV endocardium rather vertically from the opposite direction [10]. This difference in myocardial sheets orientation at end-diastole is considered to cause the difference in systolic radial strain between IVS and LVPW. Similarly, in the present study, both radial normal strain and shear strain were significantly larger in LVPW than in IVS, suggesting “myocardial sheets” theory also holds for intact human LV (Additional files 1 and 2). Compared to other speckle tracking techniques, the current method allows verifying the “myocardial sheets” theory. Shear strain seems to vary from base to apex and is negative at basal septum might mean that shear strain might play an important role in maintaining the rotation and torsion of LV and contribute to the LV systolic function.

Traditional M-mode is also at a high temporal and spatial resolution and allows the definition of regional myocardial thickening/thinning parameters. However, the normal strain based on 2D speckle tracking technique allows the study of both regional radial thickening and thinning, and regional longitudinal and circumferential shortening and lengthening, and thus can measure three aspects of regional function as opposed to only the radial parameters measured by traditional grey-scale M-mode. Although anatomical M-mode can measure the wall thickening at multiple places, it is still restricted to one aspect. Besides, the traditional M-mode does not allow the shear strain analysis.

### Physiological dyssynchrony

The 2D speckle tracking technique (Aloka Co., Japan) used in the present study showed that time to peak radial strain could be used to assess the intra and inter-ventricular synchrony. However, in most other studies, measured strain or strain rate were acquired by averaging the data from multiple points throughout the target segment, which might mask the true myocardial mechanical properties of the individual segments, rendering that the myocardial strains seem to reach the peak at the same time in normal hearts [1-8]. However, the study by Zwanenburg et al. showed that the time to peak systolic strain of each segment was not equal among LV segments by MRI tagging, raising the possibility that there are pitfalls of some of 2D echo speckle tracking system [15].

Recently, a number of indexes of intra and inter-ventricular dyssynchrony were used to differentiate responders to cardiac resynchronization therapy (CRT) from non-responders [16-18]. Intraventricular asynchrony was evaluated by calculating the septal-to-posterior wall motion delay (SPWMD) as the shortest time interval between the maximal posterior displacement of the septum and the posterior wall. Pitzalis et al. demonstrated that an SPWMD ≥ 130 ms can prospectively identify the candidates for cardiac resynchronization therapy (CRT) [16], which has been used extensively over the last years in the therapeutic management of patients with end-stage heart failure. However, Mele D et al. found that the septal-to-posterior wall thickening delay (SPWTD) differentiated responders from non-responders to CRT with better accuracy and reproducibility compared with SPWMD. They recommended that time to maximal thickening (not just wall motion) should be measured when applying the M-mode approach for evaluation of LV dyssynchrony [17].

Our study showed that the peak radial normal strain delayed in LVPW than in IVS even in healthy human subjects. It demonstrated that the time to peak radial normal strain was shortest at the base of IVS, and then increased progressively from the mid to the apical of IVS to the apical, mid and base of LVPW. This peak time difference may be explained by the direction of electrical conduction. Note that when employing the index of time to peak radial normal strain for patient selection and assessment of CRT. When these indexes were utilized, the presence of physiological dyssynchrony as shown in this study should be taken into consideration.

### Limitations

In this study, there is no analysis of differences at the three levels within the same wall. Instead, we focused on the comparison of the strains that occurred in different wall (IVS and LVPW) at the same LV level since the main purpose of this study was to verify the myocardial sheets theory in the human hearts. For the same reason, only two walls (IVS and LVPW) are considered in this study although this system allows the evaluation of six myocardial points from a single LV short-axis view.

There are some technical limitations. The first is that speckle tracking echocardiography is dependent on frame rates, as well as image resolution. The second is that the user interface required for the endocardial border tracing, and care must be taken to manually fine-tune the region of interest. The third is that speckle tracking echocardiography is two-dimension, out-of-plane movement and the reproducibility could also be the factors that influence the accuracy of this technique. In the present study, high frame rate was acquired and only the subjects with high quality cardiac images were adopted for analysis. Further studies with MRI validation need to be done. In addition, the present study that covered a group of young healthy subjects does not apply to the entire populations, and a separate study covered patients with heart diseases was being performed and the data were not included in the present study. In addition, the superiority of these new parameters over classic longitudinal, circumferential and radial strain in clinical practice needs to be further studied.

## Conclusions

The present Two-Dimensional Echocardiographic Speckle Tracking System can be used to assess cardiac mechanics from different perspectives and the data from the current study may be used as reference value. The findings of peak systolic radial normal strain of LVPW was much greater than that of IVS and occurred much later, and LVPW showed different shear strain on LV long-axis view from that of IVS, indicated that “myocardial sheets” theory also holds for intact human LV. In addition, the physiological dyssynchrony existed in healthy human subjects was quantified, which should be considered when evaluating the diseased hearts by this technique.

## Abbreviations

LV: Left ventricular; IVS: Interventricular septum; LVPW: Left ventricular posterior wall; 2-D: Two-dimensional; LVFW: Left ventricular free walls; EF: Ejection fraction; FS: Fractional shortening; E/A: Early peak flow velocity/atrial peak flow velocity across the transmitral valve; CRT: Cardiac resynchronization therapy; SPWMD: Septal-to-posterior wall motion delay; SPWTD: Septal-to-posterior wall thickening delay; LAX: Long-axis view of left ventricle; SAX: Short-axis view of left ventricle.

## Competing interests

The authors declare that they have no competing interests.

## Authors’ contributions

YLJ carried out the echocardiographic data acquisition, analysis and interpretation, drafted the manuscript and gave final approval of the version to be published. TK participated in the study conception and design, analysis and interpretation of data, revising the manuscript and gave the final approval of the version to be published. UK, EA, SK, KT, SM and NR substantially contributed to analysis and interpretation of data, and gave final approval of the version to be published.

## Supplementary Material

Additional file 1 **Animation illustrating the myocardial sheets motion proposed by LeGrice et al. [**[2]**].** On the cross-section of LV, myocardial sheets with the width of 4 myocytes separated by cleavage planes of loose connective tissue could be identified. When myocardial cells contract, the myocardial sheets slide on each other causing shear strain. The myocardial sheets approached the LV endocardium obliquely from the apical direction, becoming nearly parallel to the endocardial surface in LVPW while, in the IVS, the sheets approached the LV endocardium rather vertically from the opposite direction. This difference in sheets orientation at end-diastole would cause difference in systolic radial strain between IVS and LVPW. Myocardial sheets slide relative to each other and change orientation from end diastole to end systole, and this sliding (shearing) motion pushes the endocardium towards the LV cavity, which is more prominent in LVPW than IVS. As a result, both radial normal strain and shear strain are significantly larger in LVPW than in IVS. Click here for file

Additional file 2 **Measurements of radial normal strain of IVS and LVPW at mid level in parasternal LV long axis view (corresponding to Figure **2**).** Right panel shows radial normal strain curves for left ventricular posterior wall (LVPW) and interventricular septum (IVS). IVS strain peaks is earlier than LVPW strain, and peak strain value is about 2 times larger in LVPW than in IVS. Click here for file
